# A 10-second fluid challenge guided by transthoracic echocardiography can predict fluid responsiveness

**DOI:** 10.1186/cc13891

**Published:** 2014-05-27

**Authors:** Yunfan Wu, Shusheng Zhou, Zhihua Zhou, Bao Liu

**Affiliations:** 1 Department of Critical Care Medicine, Affiliated Provincial Hospital of Anhui Medical University, No. 17, Lujiang Road, Hefei 230001, Anhui Province, China; 2 Department of Critical Care Medicine, Affiliated Hospital of Neurology Institute, Anhui University of Traditional Chinese Medicine, No. 357, Yangtze river middle road, Hefei 230061, Anhui Province, China

## Abstract

**Introduction:**

The accurate assessment of intravascular volume status for the therapy of severe hypovolemia and shock is difficult and critical to critically ill patients. Non-invasive evaluation of fluid responsiveness by the rapid infusion of a very limited amount of volume is an important clinical goal. This study aimed to test whether echocardiographic parameters could predict fluid responsiveness in critically ill patients following a low-volume (50-ml crystalloid solution) infusion over 10 seconds.

**Methods:**

We prospectively studied 55 mechanically ventilated patients. Echocardiography was performed during a 50-ml infusion of crystalloid solution over 10 seconds and a further 450 ml over 15 minutes. Cardiac output (CO), stroke volume (SV), aortic velocity time index (VTI), and left ventricular ejection fraction (LVEF) were recorded. Patients were classified as responders (Rs) if CO increased by at least 15% following the 500-ml volume expansion or were classified as non-responders (NRs) if CO increased by less than 15%. Area under the receiver operating characteristic curves (AUC) compared CO variations after 50 ml over 10 seconds (∆CO50) and 500 ml over 15 minutes (∆CO500) and the variation of VTI after infusion of 50 ml of fluid over 10 seconds (∆VTI50).

**Results:**

In total, 50 patients were enrolled, and 27 (54%) of them were Rs. General characteristics, LVEF, heart rate, and central venous pressure were similar between Rs and NRs. In the Rs group, the AUC for ∆CO50 was 0.95 ± 0.03 (*P* <0.01; best cutoff value, 6%; sensitivity, 93%; specificity, 91%). Moreover, ∆CO50 and ∆CO500 were strongly correlated (*r* = 0.87; *P* <0.01). The AUC for ∆VTI50 was 0.91 ± 0.04 (*P* <0.01; best cutoff value, 9%; sensitivity, 74%; specificity, 95%). ∆VTI50 and ∆CO500 were positively correlated (*r* = 0.72; *P* <0.01).

**Conclusion:**

In critically ill patients, the variation of CO and VTI after the administration of 50-ml crystalloid solution over 10 seconds (∆CO50 and ∆VTI50) can accurately predict fluid responsiveness.

**Trial registration:**

Current Controlled Trials ISRCTN10524328. Registered 12 December 2013.

## Introduction

Assessment of intravascular volume status is difficult in critically ill patients. Evidence suggests that only 50% of hemodynamically unstable patients respond to a fluid challenge [1,2]. Moreover, if cardiopulmonary function cannot compensate for the increase in preload, fluid loading may compromise microvascular perfusion and oxygen delivery and cause or aggravate peripheral and pulmonary edema [3,4]. Therefore, inappropriate fluid expansion can increase morbidity and mortality [5-7], making it important to accurately assess fluid responsiveness in critically ill patients [8,9].

Research has suggested that volume responsiveness can be defined as a 15% increase in stroke volume (SV) or cardiac output (CO) after a 500-ml infusion [10-12]. Over the last decade, multiple studies have demonstrated that static parameters have limited the predictive value for fluid responsiveness. These include pulmonary artery occlusion pressure, right ventricular end-diastolic volume, left ventricular end-diastolic volume, central venous pressure (CVP), and inferior vena cava (IVC) diameter [2,13-15]. Furthermore, dynamic parameters have been extensively studied, leading to greater clinical use, including respiratory changes in aortic blood velocity, superior vena cava collapsibility, IVC collapsibility, and changes in SV and CO due to passive leg raising (PLR) [14,16-19]. In addition, many studies have demonstrated that echocardiography [2,16,18,20] offers a non-invasive, dynamic, and qualitative assessment of volume responsiveness in patients with hemodynamic failure, such as changes in the velocity time integral.

The novel, mini-fluid challenge technique proposed by Muller *et al.*[21] demonstrated that a fluid challenge response can be evaluated by using small fluid volumes (100 ml of hydroxyethyl starch) over 1 minute. We hypothesized that a small fluid preload and faster infusion rate can also predict responsiveness. This study aimed to determine whether echocardiographic parameters following a 50-ml infusion of crystalloid solution over 10 seconds can predict fluid responsiveness in critical ill patients with hypovolemic or septic shock.

## Methods

### Patients

Mechanically ventilated adults admitted to the intensive care unit (ICU) with hypovolemic shock, severe sepsis, or septic shock were included from March to October 2013. All patients or their families signed consent forms to participate in this study. The medical ethics committee of Anhui Provincial Hospital approved the protocol.

Patients were required to have at least one sign of inadequate tissue perfusion [22]: acute circulatory failure defined as a systolic arterial pressure of 90 mm Hg (or a decrease of 40 mm Hg in a patient with hypertension), urine output of below 0.5 ml/kg per hour for over 1 hour, tachycardia (heart rate (HR) of greater than 100 beats per minute), and mottled skin. Clinical diseases were associated with systemic inflammatory response syndrome, septic shock, and controlled massive hemorrhage. The attending physician clinically determined the need for volume expansion (VE). If patients were treated with norepinephrine, the dose remained unchanged from before VE until all hemodynamic measurements were complete. Exclusion criteria were the following: age of less than 18 years, moribund, cardiomyopathy, pulmonary edema, increased intracranial pressure, pregnancy, active bleeding, atrial fibrillation, cardiac arrhythmias, and myocardial ischemia or infarction within 1 month before the study.

### Measurements

**Figure 1:**
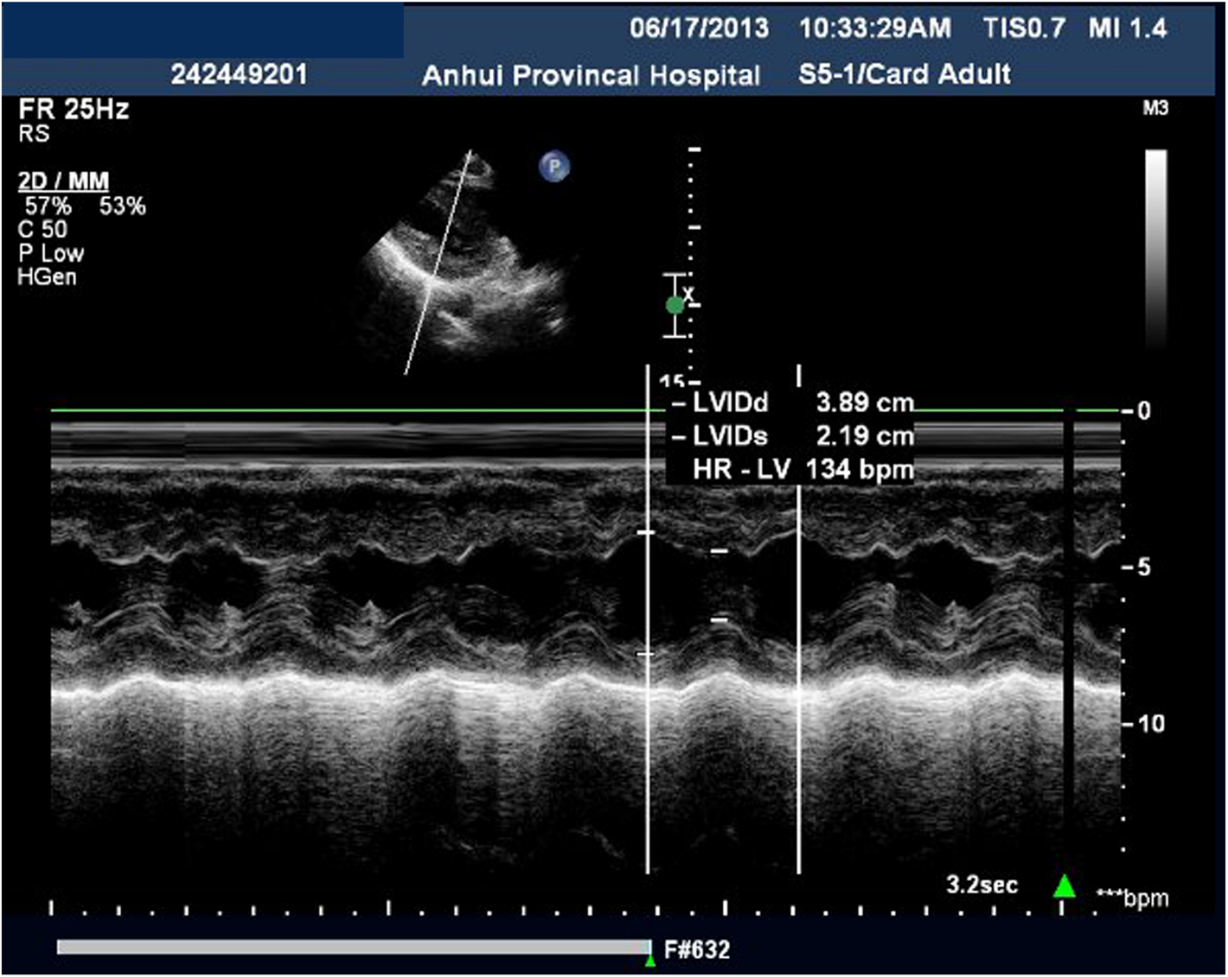
Photo of an echocardiographic Doppler flow velocity measurement from the level of the aortic annulus from the parasternal long-axis window.

Echocardiographic parameters were measured by using a bedside Philips IU22 xMATRIX ultrasound system (Royal Philips Electronics, Amsterdam, The Netherlands) with a 3- to 5-MHz phased-array probe. M-mode (time-motion) echocardiography was employed at the level of the aortic annulus in a two-dimensional view from the parasternal long-axis window (Figure 1). We measured the aortic diameter (D), left ventricular end-diastolic diameter, left ventricular end-systolic diameter, and HR. Using the in-built software, we determined the left ventricular end-diastolic volume (LEDV) and left ventricular end-systolic volume (LESV). In addition, HR, blood pressure, CVP, and other hemodynamic parameters were recorded throughout the study. The following equations were used to calculate additional parameters: SV = [LEDV – LESV]; left ventricular ejection fraction (LVEF, %) = [(SV ÷ LEDV) × 100]; and CO = [SV × HR]. Aortic blood flow and velocity time integral (VTI) were obtained from an apical five-chamber view.

### Fluid responsiveness

A 50-ml bolus of crystalloid solution was given to the patient over 10 seconds through an intrathoracic central venous catheter. VTI was recorded during and immediately after bolus administration. The remaining 450 ml of crystalloid solution was infused at a constant rate over 15 minutes, and the VTI was measured again. The mean echocardiographic measurements over three consecutive respiratory cycles were recorded, and parameters were analyzed by using frozen images from M-mode recordings. Changes in parameters (HR, CVP, VTI, SV, and CO) were measured after the 50-ml infusion over 15 seconds and after the full 500 ml over 15 minutes; these were referred to as ∆[parameter]50 and ∆[parameter]500, respectively. Patients with an increase in CO after a 500-ml infusion over 15 minutes (∆CO500) of 15% or more were classified as responders (Rs), and those without an increase were classified as non-responders (NRs) [23]. An experienced ultrasound physician performed all echocardiographic collection, but all measurements were conducted by another physician, who was blinded to the clinical diagnosis.

### Statistical analysis

The SPSS 16.0 software (SPSS Inc., Chicago, IL, USA) was used for statistical analyses. Normality was tested by using the Shapiro-Wilk test, and comparisons of hemodynamic parameters between Rs and NRs were assessed by using the Mann–Whitney *U* test. The Friedman test and Wilcoxon rank-sum test were used to detect changes over time (baseline, after 50 ml, and after 500 ml) within the same group. Linear correlations were tested by using the Spearman rank correlation and Pearson tests for non-normal and normal distributions, respectively. Receiver operating characteristic (ROC) curves were constructed to evaluate the ability of each parameter to predict fluid responsiveness after VE by varying the discriminating threshold of each measurement. The area under each ROC curve (AUC) was calculated and expressed as AUC ± standard deviation (SD). Cutoff values were chosen with the highest Youden index, calculated as [1 - (1 - sensitivity) - (1 - specificity)]. Probability (*P*) values of less than 0.05 were considered statistically significant. Data are presented as mean ± SD if normally distributed or as median (interquartile range) if not normally distributed.

## Results

In total, 55 patients were included; 5 patients were excluded because adequate images could not be obtained. Table 1 summarizes the characteristics of the 50 patients with a comparison between Rs (n = 27) and NRs (n = 23). Diagnoses were severe sepsis or septic shock (n = 36), severe trauma (n = 10), gastrointestinal bleeding (n = 3), and dehydration (n = 1). At baseline, general characteristics, HR (*P* = 0.32), LVEF (*P* = 0.33), and CVP (*P* = 0.40) were similar between the two groups. Significant differences in the following were predictive of fluid responsiveness: systolic blood pressure (SBP), VTI, SV, and CO. The last three parameters were clearly lower among Rs than NRs at baseline.

**Table 1:** Patient characteristics stratified by fluid responders and non-responders at baseline

**Characteristics**	**All participants (n = 50)**	**Responders (n = 27)**	**Non-responders (n = 23)**	***P*** **value**
General characteristics				
Age, years	60 ± 17	61 ± 15	59 ± 18	0.50
APACHE II score	23 ± 4	24 ± 4	22 ± 4	0.10
Sex, male/female	34/16	19/8	15/8	0.70
Vital signs				
HR, beats/min^a^	89 (68–100)	89 (68–116)	88 (64–95)	0.32
SBP, mm Hg	92 ± 21	86 ± 23	99 ± 16	0.04
CVP, mm Hg^a^	5 (4–6)	5 (4–6)	5 (5–7)	0.40
Echocardiographic measurements				
LVEF, %	61.1 ± 11.9	62.2 ± 13.8	60.0 ± 9.5	0.33
VTI, cm	30.9 ± 12.8	25.6 ± 13.8	37.1 ± 8.1	<0.01
SV, ml^a^	78.8 (50.8-97.4)	66.7 (39.5-83.1)	83.1 (74.1-119.7)	<0.01
CO, l/min^a^	8.2 (6.0-12.7)	7.2 (5.5-9.7)	9.7 (7.0-15.5)	0.05

Values are expressed as number (percentage) or as mean ± standard deviation. Percentages are rounded, so the total may not equal 100%. ^a^Values are expressed as median (interquartile range). APACHE, Acute Physiology and Chronic Health Evaluation; CO, cardiac output; CVP, central venous pressure; HR, heart rate; LVEF, left ventricular ejection fraction; SBP, systolic blood pressure; SV, stroke volume; VTI, aortic velocity time index.

Table 2 shows that the hemodynamic parameters are significantly different between the Rs and NRs groups after 50 and 500 ml VE (including HR, SBP, VTI, SV, and CO). The Wilcoxon rank-sum test for hemodynamic parameters at baseline, post-50 ml, and post-500 ml VE was statistically significant, excluding HR between the baseline and after 50 ml VE in Rs (*P* = 0.41) and NRs (*P* = 0.18).

**Table 2:** Hemodynamic variables were measured at baseline, during volume expansion

	**Baseline**	**50 ml VE**	**500 ml VE**	***P*** **value**
HR, beats/min				
Responders	92 ± 28	91 ± 26	87 ± 27	<0.01
Non-responders	83 ± 17	83 ± 18	79 ± 17	<0.01
SBP, mm Hg				
Responders	86 ± 23	87 ± 22	96 ± 21	<0.01
Non-responders	99 ± 16	102 ± 17	105 ± 19	<0.01
LVEF, %				
Responders	62.2 ± 13.8	63.6 ± 11.6	64.5 ± 11.5	0.12
Non-responders	60.0 ± 9.5	62.4 ± 10.8	63.0 ± 7.7	0.06
VTI, cm				
Responders	25.6 ± 13.8	28.7 ± 15.2	34.4 ± 17.7	<0.01
Non-responders	37.1 ± 8.1	38.2 ± 8.5	40.7 ± 9.0	<0.01
SV, ml				
Responders	65.6 ± 26.0	76.1 ± 29.4	93.8 ± 39.4	<0.01
Non-responders	93.3 ± 26.7	97.1 ± 27.6	101.7 ± 28.8	<0.01
CO, l/min				
Responders	8.4 ± 4.0	9.3 ± 4.2	10.7 ± 4.5	<0.01
Non-responders	10.7 ± 4.3	10.9 ± 4.4	11.3 ± 4.5	<0.01

Values other than *P* values are expressed as mean ± standard deviation. CO, cardiac output; HR, heart rate; LVEF, left ventricular ejection fraction; SBP, systolic blood pressure; SV, stroke volume; VE, volume expansion; VTI, aortic velocity time index.

**Figure 2:**
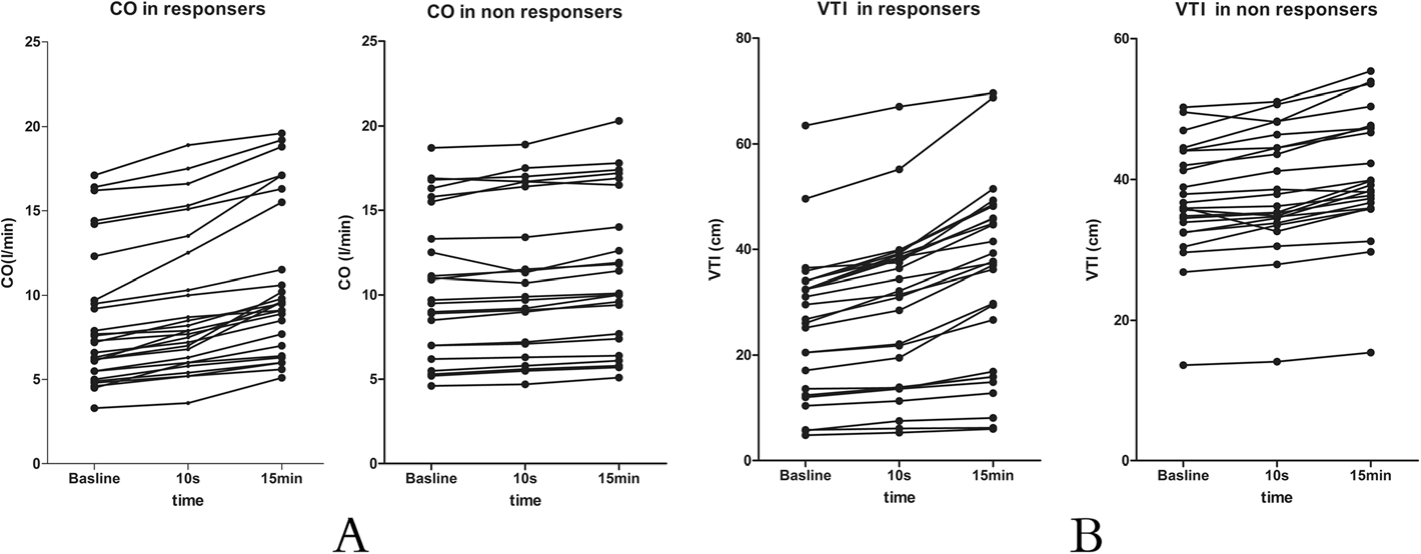
Individual values for cardiac output (CO) (A) and aortic velocity time index (VTI) (B) at baseline, 10 seconds, and 15 minutes in responders and non-responders.

**Figure 3:**
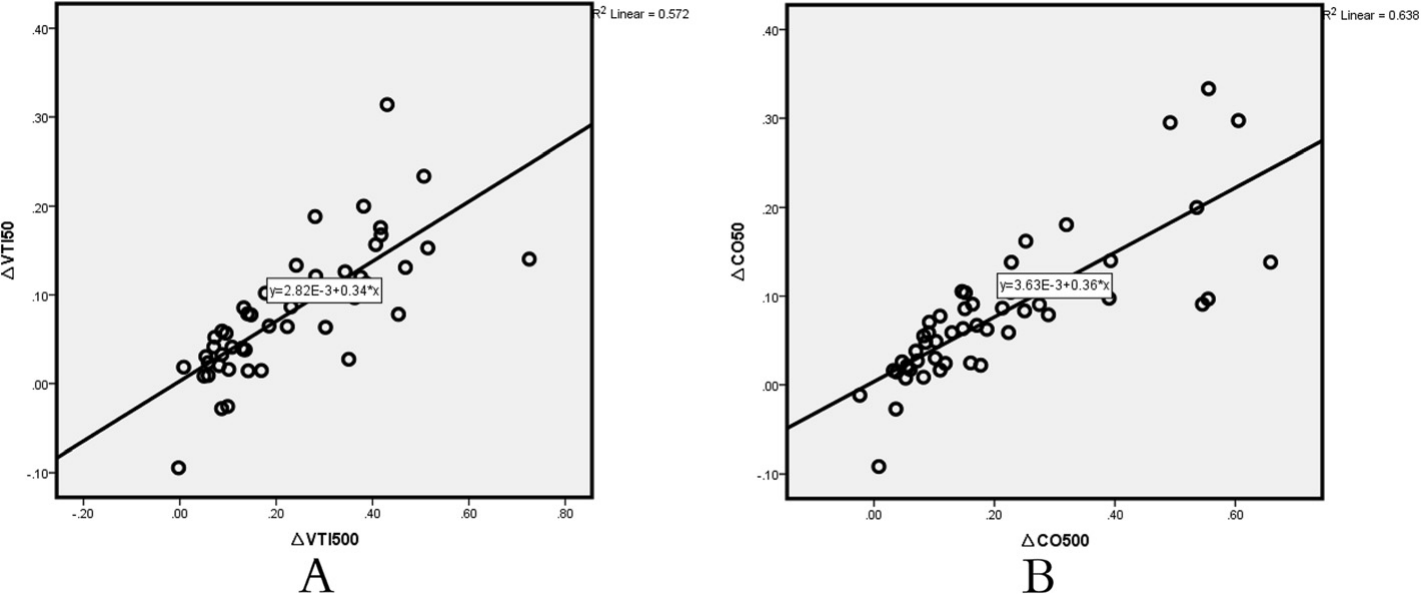
**(A) Correlation between ∆VTI50 (%) and ∆VTI500 (%).(B)** Correlation between ∆CO50 (%) and ∆CO500 (%). ∆CO50, variation of cardiac output after infusion of 50 ml of fluid over 10 seconds; ∆CO500, variation of cardiac output after infusion of 500 ml fluid over 15 minutes; ∆VTI50, variation of velocity time index after infusion of 50 ml of fluid over 10 seconds; ∆VTI500, variation of velocity time index after infusion of 500 ml of fluid over 15 minutes.

**Figure 4:**
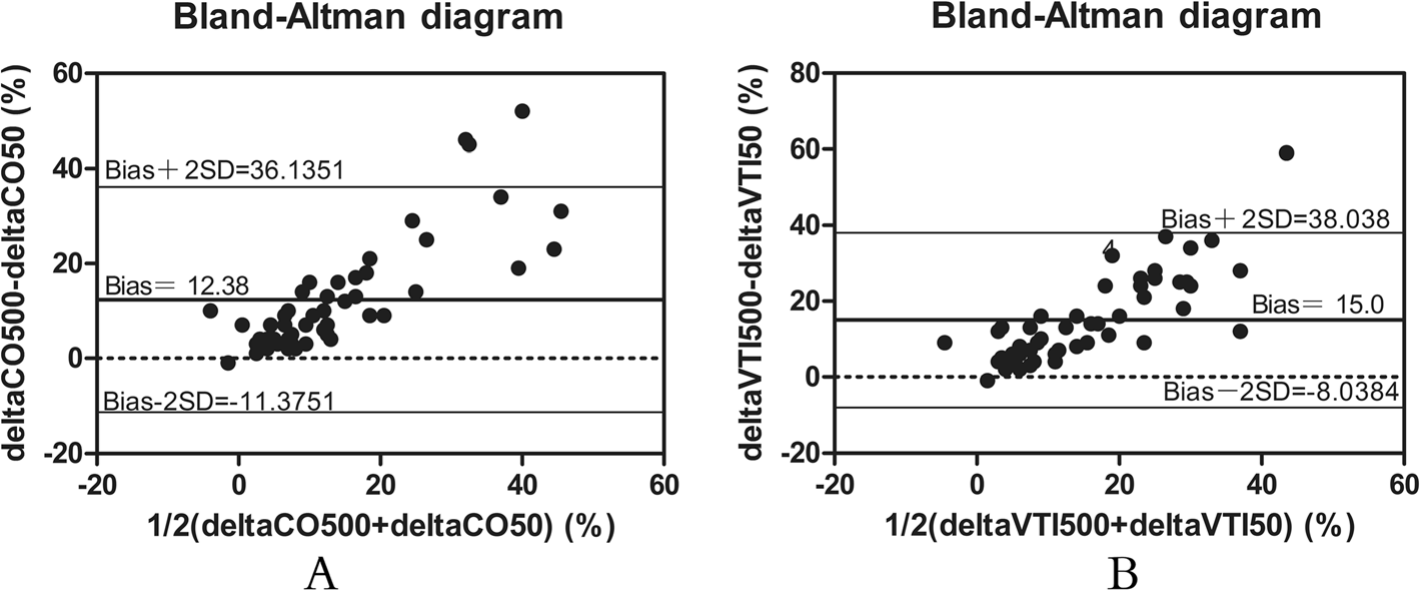
**Bland and Altman diagram between variation of cardiac output (A) and variation of velocity time index (B) after 50- or 500-ml volume expansion.** ∆CO50, variation of cardiac output after infusion of 50 ml of fluid over 10 seconds. ∆CO500, variation of cardiac output after infusion of 500 ml of fluid over 15 minutes; ∆VTI50, variation of velocity time index after infusion of 50 ml of fluid over 10 seconds. ∆VTI500, variation of velocity time index after infusion of 500 ml of fluid over 15 minutes; CO, cardiac output; VTI, aortic velocity time index.

Figure 2 shows that individual changes in CO and VTI produced by VE at baseline, 10 seconds, and 15 minutes in the two groups. Correlations were tested by using the Spearman rank correlation (Figure 3). In all patients, ∆VTI50 was positively and significantly correlated with ∆VTI500 (*r* = 0.83; *P* <0.01) (Figure 3A). Similarly, ∆SV50 strongly and significantly correlated with ∆SV500, as did ∆CO50 and ∆CO500 (*r* = 0.90; *P* <0.01) (Figure 3B). Moreover, ∆VTI50 and ∆CO500 were positively correlated (*r* = 0.72; *P* <0.01). The Bland-Altman plot presenting differences between ∆CO50 and ∆CO500 is given in Figure 4A. The bias was 12%. The 95% limits of agreement (LOAs) were (-11%, 36%). Figure 4B shows the Bland-Altman plots of the difference in ∆VTI50 versus ∆VTI500. The bias was 15%. The 95% LOAs were (-8%, 38%).

**Figure 5:**
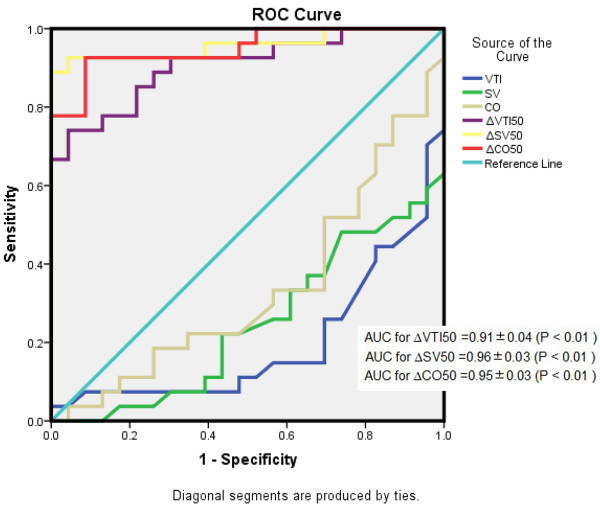
**Receiver operator characteristic (ROC) curves for ∆VTI50 (%), ∆SV50 (%), and ∆CO50 (%) after infusion of 50 ml of fluid over 10 seconds, and ROC curves of VTI (cm), SV (ml), and CO (l/min) measured at baseline.** ∆CO50, variation of cardiac output after infusion of 50 ml of fluid over 10 seconds; ∆SV50, variation of stroke volume after infusion of 50 ml of fluid over 10 seconds; ∆VTI50, variation of velocity time index after infusion of 50 ml of fluid over 10 seconds; CO, cardiac output; SV, stroke volume; VTI, aortic velocity time index. Diagonal segments are produced by ties.

As shown in Figure 5, the AUCs for ∆VTI50, ∆SV50, and ∆CO50 suggest that all accurately predicted fluid responsiveness after infusion of 50-ml VE over 10 seconds. The AUCs for ∆VTI50, ∆SV50, and ∆CO50 were 0.91 ± 0.04 (*P* <0.01), 0.96 ± 0.03 (*P* <0.01), and 0.95 ± 0.03 (*P* <0.01), respectively. The AUCs for VTI, SV, and CO at baseline had lower accuracy for predicting fluid responsiveness compared with ∆VTI50 and ∆CO50.

**Figure 6:**
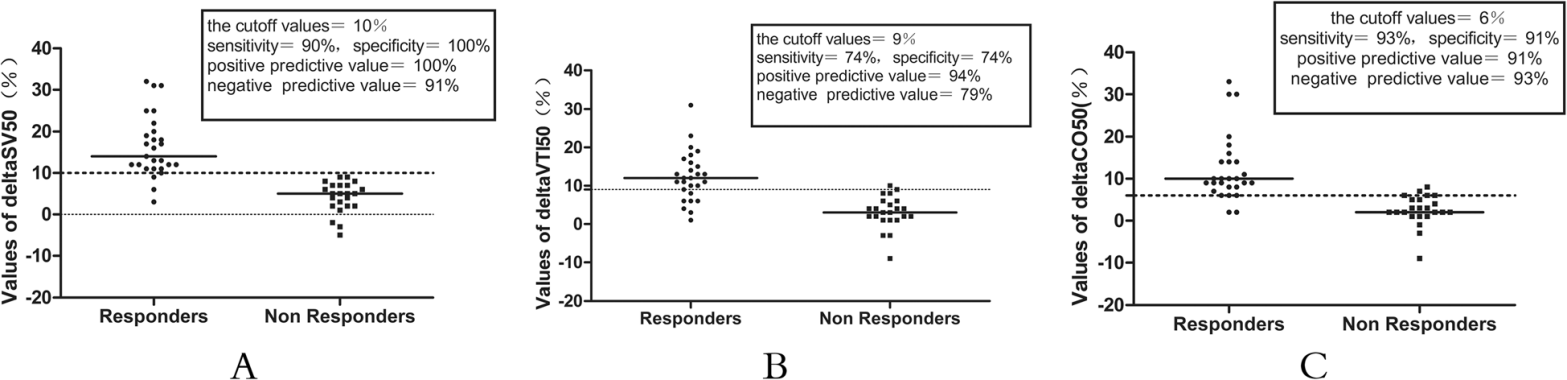
**Individual values of ∆VTI50 (%) (A), ∆SV50 (%) (B), and ∆CO50 (%) (C) after infusion of 50 ml of fluid over 10 seconds changed in patients with volume expansion-induced changes in stroke volume (SV) of at least 15% (responders) and less than 15% (non-responders).** ∆CO50, variation of cardiac output after infusion of 50 ml of fluid over 10 seconds; ∆SV50, variation of stroke volume after infusion of 50 ml of fluid over 10 seconds; ∆VTI50, variation of velocity time index after infusion of 50 ml of fluid over 10 seconds; CO, cardiac output; VTI, aortic velocity time index.

Table 3 and Figure 6 showed that, for ∆VTI50 after infusion of 50-ml VE over 10 seconds, the optimum cutoff value was 9% (sensitivity, 74%; specificity, 95%). The best AUC cutoff values when predicting fluid responsiveness were 10% for ∆SV50 (sensitivity, 90%; specificity, 100%) and 6% for ∆CO50 (sensitivity, 93%; specificity, 91%), respectively. After 500-ml VE, the best cutoff value was 19% for ∆VTI500 (sensitivity, 89%; specificity, 100%).

**Table 3:** Accuracy of echocardiographic parameters for predicting fluid responsiveness

	**AUC**	**Cutoff values**	**Sensitivity**	**Specificity**	**Positive predictive value**	**Negative predictive value**
∆VTI50	0.91 ± 0.04	9%	74%	95%	94%	79%
∆VTI500	0.95 ± 0.03	19%	89%	100%	100%	90%
∆CO50	0.95 ± 0.03	6%	93%	91%	91%	93%
∆SV50	0.96 ± 0.03	10%	90%	100%	100%	91%
∆SV500	1.00	15%	100%	100%	100%	100%

∆CO50, variation of cardiac output after infusion of 50 ml of fluid over 10 seconds; ∆SV50, variation of stroke volume after infusion of 50 ml of fluid over 10 seconds; ∆SV500, variation of stroke volume after infusion of 500 ml fluid over 15 minutes; ∆VTI50, variation of velocity time index after infusion of 50 ml of fluid over 10 seconds; ∆VTI500, variation of velocity time index after infusion of 500 ml of fluid over 15 minutes; AUC, area under the receiver operating characteristic curves.

## Discussion

Fluid resuscitation is the cornerstone of hypovolemic shock, severe sepsis, or septic shock patient management. However, when patients are on the relatively flat part of the Frank-Starling curve, preload VE can be deleterious. Consequently, monitoring volume responsiveness has become increasingly important in the ICU.

Pleural pressure, intra-abdominal pressures, mechanical ventilation, capillary permeability, and other factors can influence CVP to predict fluid responsiveness. Previous studies [13,14,24,25] have demonstrated that baseline CVP has a poor ability of predicting fluid responsiveness in critically ill patients. Our study also demonstrated that the initial CVP was not different between the two groups and the AUC for initial CVP was low. Moreover, consistent with earlier research [25], the present study found that baseline HR was inadequate for predicting fluid responsiveness. Interestingly, there was no statistically significant for HR between baseline and after 50-ml VE in Rs (*P* = 0.41) and NRs (*P* = 0.18). This means that crystalloid distributed rapidly through the extracellular compartment, which did not affect sympathetic stimulation or cardiac reflexes. Our study suggested that there was a significant difference in SBP between Rs and NRs. However, the literature suggests that SBP does not reliably predict a patient’s response to fluid administration, which was affected by drugs, stress, pain, fever, anemia, intrinsic heart disease, and many other factors [26].

Recently, dynamic echocardiographic parameters ∆SV, ∆CO, and ∆VTI induced by VE or PLR have gained increased attention; moreover, VE and PLR can reliably predict fluid responsiveness regardless of the ventilation mode or underlying cardiac rhythm in the majority of ICU patients [18,27-30]. However, PLR is susceptible to the effects of increased intra-abdominal pressure and cannot be used in patients with fracture or following surgery [31,32]. Because of such limitations, a more secure and convenient method of predicting fluid response is required to reduce unnecessary medical intervention. In echocardiography, SV was also determined as VTI × [π (D^2^) ÷ 4]. With D being constant in a given patient, VTI and SV changes are expected to be equal. Over the past decade, a number of reports have correlated ∆VTI with fluid responsiveness [18,29,33-35]. In our study, the ∆VTI500 (AUC, 0.95 ± 0.03) had a high accuracy for predicting fluid responsiveness.

As demonstrated by the Frank-Starling curve, the beginning of the fluid challenge induces a more significant increase in stroke volume on the steep portion of the curve because of a preload reserve in the left ventricular function. Muller *et al.*[21] proposed the mini-fluid challenge technique and demonstrated that a fluid challenge response can be evaluated by using small fluid volumes. Inspired by this, we hypothesized that a 50-ml infusion of crystalloid over 10 seconds could predict fluid responsiveness. Although this fluid regime was arbitrarily chosen, it was based on the peak increase in intravascular volume with lactated crystalloid solution occurring immediately after completion of the rapid fluid infusion [36,37] and the low clinical risk of crystalloid solution [22,38].

In the present study, the relatively high correlation coefficient between ∆VTI50 and ∆VTI500 was *r* = 0.83 (*P* <0.01) and the correlation between ∆CO50 and ∆CO500 was *r* = 0.90 (*P* <0.01), respectively. Moreover, Bland-Altman-like analysis of the same data indicated that there was a good concordance for two measurements. This was associated with a trend that a higher VE can lead to a higher change of CO.

The ROC analysis of echocardiographic parameters revealed that a response to 50-ml crystalloid bolus over 10 seconds enables accurate prediction of fluid responsiveness, particularly ∆VTI50 and ∆CO50. The AUCs for these echocardiographic parameters were above 0.90. The best AUC cutoff for ∆CO50 when predicting fluid responsiveness was 6% (sensitivity, 93%; specificity, 91%), suggesting that a mini-fluid challenge of 50-ml crystalloid bolus over 10 seconds enables accurate bedside prediction of a 15% increase in ∆CO500 in 27 adults. In Figure 5, we also proved that these dynamic echocardiographic indices after 50-ml VE had greater predictive value for fluid responsiveness than static echocardiographic indices (i.e., those measured at baseline). Above all, our data support the hypothesis that the response to a mini-fluid challenge can predict responsiveness to a larger challenge. This means that a low fluid volume can be used to predict responsiveness and decrease the deleterious effect of an unnecessary fluid infusion in NRs. In our study, ∆CO50 had higher statistical accuracy than ∆VTI50. However, echocardiography measured VTI only with pulsed Doppler from the apical five-chamber view, which means that VTI was quicker and easier to perform than CO. Multiple studies have considered that ∆VTI can accurately and sensitively predict fluid responsiveness [18,29,33,35,39].

This present study has several limitations. First, the patient sample was small and may limit the external validity. Second, because our study excluded patients with cardiac arrhythmias, we do not know its applicability in this setting. Third, transthoracic echocardiography has many specific limitations: It is technically demanding, and many ICUs may lack suitably skilled operators. The sharpness of ultrasound images will be affected by obesity, chest deformity, high abdominal pressure, patient positioning, and other factors. Fourth, we did not assess the impact of measuring time. This assessment deserves further studies.

## Conclusions

In contrast to the study by Muller *et al*. [21], the present research had shown that even small fluid challenges over shorter periods can be sufficient in predicting fluid responsiveness among critically ill patients. In conclusion, we believe our study provides new insights into effective prediction of fluid responsiveness by ∆CO50 and ∆VTI50 after 50-ml crystal solution over 10 seconds.

## Key messages

• Echocardiography offers a convenient, non-invasive, dynamic, and qualitative assessment of volume responsiveness in the ICU.

• By using a smaller fluid volume and faster infusion rate than ever before, the study wanted to find a relatively accurate and sensitive fluid challenge technique with more decrease morbidity and mortality.

• The variation of CO and VTI measured by transthoracic echocardiography after the administration of 50-ml crystalloid solution over 10 seconds is able to reliably predict fluid responsiveness among critically ill patients.

• Despite a lower sensitivity and several limitations, the mini-fluid challenge technique may provide a new research perspective for effective and rapid prediction of fluid responsiveness in the future.

## Abbreviations

∆: Delta; ∆CO50: variation of cardiac output after infusion of 50 ml of fluid over 10 seconds; ∆CO500: variation of cardiac output after infusion of 500 ml of fluid over 15 minutes; ∆SV50: variation of stroke volume after infusion of 50 ml of fluid over 10 seconds; ∆SV500: variation of stroke volume after infusion of 500 ml of fluid over 15 minutes; ∆VTI50: variation of velocity time index after infusion of 50 ml of fluid over 10 seconds; ∆VTI500: variation of velocity time index after infusion of 500 ml of fluid over 15 minutes; AUC: area under the receiver operating characteristic curves; CO: cardiac output; CVP: central venous pressure; D: aortic diameter; HR: heart rate; ICU: intensive care unit; IVC: inferior vena cava; LEDV: left ventricular end-diastolic volume; LESV: left ventricular end-systolic volume; LOA: limit of agreement; LVEF: left ventricular ejection fraction; NR: non-responder; PLR: passive leg raising; R: responder; ROC: receiver operating characteristic; SBP: systolic blood pressure; SD: standard deviation; SV: stroke volume; VE: volume expansion; VTI: aortic velocity time index.

## Competing interests

The authors declare that they have no competing interests.

## Authors’ contributions

YW performed the data collection and drafted the manuscript. ZZ performed the statistical analysis and assisted in the drafting of the manuscript. SZ and BL conceived of the study, participated in its design, and coordinated the study. All authors read, revised, and approved the final manuscript.

## Authors’ information

YW, SZ and BL work at the Department of Critical Care Medicine, Affiliated Provincial Hospital of Anhui Medical University. ZZ works at the Department of Critical Care Medicine, Affiliated Hospital of Neurology Institute, Anhui University of Traditional Chinese Medicine.
